# The Effect of Zinc and D-Penicillamine in a Stable Human Hepatoma *ATP7B* Knockout Cell Line

**DOI:** 10.1371/journal.pone.0098809

**Published:** 2014-06-03

**Authors:** Gursimran Chandhok, Nadine Schmitt, Vanessa Sauer, Annu Aggarwal, Mohit Bhatt, Hartmut H. J. Schmidt

**Affiliations:** 1 Clinic for Transplantation Medicine, Münster University Hospital, Münster, Germany; 2 Wilson Disease Clinic, Kokilaben Dhirubhai Ambani Hospital and Medical Research Institute, Mumbai, India; University of Florida, United States of America

## Abstract

Mutations in the copper (Cu) transporter gene *ATP7B*, the primary cause of Wilson disease (WD), result in high liver Cu and death of hepatocytes. Cu chelators and zinc salts are the two most important drugs used in the treatment of WD patients; however, the molecular mechanisms of the drugs with regard to *ATP7B* expression have not been determined. A targeted knockout of *ATP7B* (KO) was established in the most widely used human hepatoma cell line, HepG2 for molecular studies of the pathogenesis and treatment of the disease. KO cells showed similar growth, Cu uptake, release, and gene expression as compared to parental cells. However, in the presence of Cu, morphological changes, oxidative stress, apoptosis, and loss of viability were observed. Induction of metallothionein (*MT1X*) after Cu exposure was significantly reduced in KO cells. Following zinc treatment, *MT1X* expression was strongly induced and a high percentage of KO cells could be rescued from Cu induced toxicity. D-penicillamine treatment had a minor effect on the viability of KO cells whereas the parental cell line showed a pronounced improvement. Combined treatment displayed a highly synergistic effect in KO cells. The data suggest that zinc has a previously unrecognized effect on the viability of hepatocytes that lack *ATP7B* due to a high induction of *MT1X* expression that compensates low gene expression after Cu exposure. A combination therapy that simultaneously targets at *MT1X* induction and Cu chelation improves the overall survival of hepatocytes for most efficient therapy of patients having WD.

## Introduction

Wilson disease (WD), an orphan disease, is caused by mutations in the ATP7B gene on chromosome 13 leading to an imbalance in copper homeostasis [1], [2]. Excessive copper (Cu) accumulation in the liver and brain are the hallmarks of this disease. The disease is manifested by liver impairment, cognitive and behavioral disturbances, movement disorders and osseomuscular symptoms [3], [4].

Cu is an essential trace element; however, if present in amounts beyond normal physiological needs, it can lead to toxicity by increasing oxidative stress and cell death [5], [6], [7]. ATP7B plays a central role in Cu homeostasis in the liver [8]. This transmembrane protein is primarily expressed in hepatocytes and mediates incorporation of Cu into ceruloplasmin and excretion of toxic Cu via bile. Impairment of ATP7B in WD leads to progressive Cu accumulation in the liver and is believed to be followed over time by spillage to other organs like brain, kidney, and cornea. Individual ATP7B mutations have been associated with various phenotypes [9].

While human hepatocytes remain the gold standard for molecular analysis of WD in the liver, availability is limited. Lower eukaryotic models, like ccc2 yeast, and mammalian cell lines, like Chinese Hamster Ovary cells (CHO), lacking ATP7B expression have proved useful in studying the functional properties of ATP7B mutants [10], [11]; however, the differences in species and organ source make it difficult to extrapolate the results to human liver. Human hepatoma cell lines are excellent cellular platforms to study ATP7B and its role in Cu homeostasis as exemplified by the most widely studied hepatic cell line, HepG2 [11], [12], [13], [14], [15], [16], [17]. Nevertheless, HepG2 and other human hepatic cell lines, like Huh7 and Hep3B, express endogenous, functional ATP7B making it difficult to study the role of ATP7B [8].

WD is treatable; however, if left untreated, it can be fatal. Frequently used drugs for treatment of WD are D-penicillamine (DPA), trientine, and zinc salts (Zn). The drugs differ in their mechanism of action, the former being Cu chelators and Zn being an inducer of antioxidant metallothionein (MT1X) in the intestine [18]. Although clinical evidence of the drugs for efficient treatment of WD have been compiled over decades, the relationship between ATP7B function and anti-copper effects of the drugs is yet to be explored at the cellular and molecular level.

In this study, we have characterized a polarized human hepatoma cell line that lacks ATP7B due to a targeted knockout mutation. The effect of Zn, DPA, and a combination of both drugs on cell viability, oxidative stress, apoptosis, gene expression, and intracellular Cu was assessed. Our results reveal that a combination of Zn and DPA is superior for increasing the viability of human hepatic cells that lack ATP7B.

## Materials and Methods

### Establishment of ATP7B knockout cell line

HepG2 (human hepatocellular carcinoma) cells were purchased from American Type Culture Collection (ATCC) and cultured in RPMI media supplemented with 10% fetal bovine serum (FBS) and 100 U/ml penicillin/streptomycin (PAA). 2.5 µg of custom made CompoZr knockout Zinc Finger Nucleases (ZFN) plasmids were transfected into HepG2 (Sigma; #CKOZFND3740-1KT). Nucleofection was performed using Amaxa Cell Line Nucleofector Kit V (Lonza; # VCA-1003). Single cell cloning in 96 well plates was performed at day 3. PCR analysis of total DNA was performed using primers 5′-CGCTCATTGAACTCTCCTCC-3′ and 5′-ACAGCAGCTTCAGGTTCAGAA-3′. PCR products were cloned into plasmid pCR2.1-TOPO (TOPO TA Cloning kit; Invitrogen). For sequence determination of ATP7B established protocols were used [19].

### Viral transduction

Lentiviral vector pRRL.PPT.SF.ATP7B.i2GFPBsd.pre expressing the coding region of human ATP7B (kind gift of A. Schambach, MHH Hannover, Germany) was used for transduction using established protocols [20]. Cells were selected in media containing 6 µg/ml blasticidin (Invitrogen).

### Western Blot analysis for ATP7B

Western blot was performed as described previously [20]. Briefly, cells were lyzed in RIPA buffer (60 mM tris-HCl, 150 mM NaCl, 2% Na-deoxycholate, 2% Triton X-10, 0.2% SDS and 15 mM EDTA) in the presence of protease inhibitors (Roche; Complete Mini, EDTA-free). Polyclonal anti-rabbit ATP7B antibody (1∶1,000) was used for detection (kind gift of I. Sandoval, Madrid, Spain). HSC70 antibody (Santa Cruz Biotechnology) staining was used as a protein loading control.

### Cumulative cell growth

3×10^5^ cells were seeded in a 6 well plate and cultured in standard cell culture medium. The cell count was determined every three days using trypan blue method and 3×10^5^ cells were reseeded in a new 6 well. The cell growth was determined over 15 days and cumulative cell count was calculated.

### Copper toxicity

10^4^ cells were seeded in triplicates in a 96 well plate and cultivated for 24 h in 100 µl DMEM medium lacking phenol red (PAA). After 48 h incubation, viability was determined by MTT (3-[4,5-dimethylthiazol-2-yl]-2,5 diphenyl tetrazolium bromide) assay as described previously.[20] For drug testing, cells were preincubated with 200 µM zinc (ZnCl_2_; AppliChem) or zinc acetate and washed twice with phosphate buffered saline (PBS; PAA) prior to Cu (CuCl_2_; Sigma Aldrich) addition. D-penicillamine (Dako) was added at 6.25 mM simultaneously with Cu. Cells were incubated for 48 h. Results were calculated as a percentage of untreated control cells (100%).

### Oxidative stress and apoptosis

OS was determined using 2′,7′-dichlorodihydrofluorescein diacetate (H_2_DCFDA; Life Technologies) staining method. 10^6^ cells were incubated with 20 µM Cu and 50 µM H_2_DCFDA dye for 30 min at 37°C and analyzed using flow cytometry (Beckman Coulter; Epics XL.MCL). For apoptosis, cells were treated with Cu for 24 h. Cells were collected and subjected to Annexin-V staining (Roche; Annexin-V-FLUOS kit).

### Real-time PCR analysis

PCR analysis was performed using primers (Table S1) as described previously [21]. Briefly, isolation of total RNA was performed by RNeasy kit (Qiagen). SuperScript II (Invitrogen) was used to transcribe 1 µg of RNA according to the instructions of the manufacturer. PCR was analyzed on the ABI Prism 7900 HT Sequence Detection System (PE Applied Biosystems). Each sample was tested in three independent experiments. Ct values were normalized to the expression of the house-keeping GAPDH gene (ΔΔct method).

### Copper determination

10^6^ cells were seeded into a 6 well plate and cultured for 12 h. The medium was replaced by medium containing 0.1 mM CuCl_2_. For chase experiments, cells were carefully washed three times with PBS and standard medium was added for another 24 h. Cells were washed thrice and the cell number was determined. Subcellular fractionation was performed as previously described [22]. Briefly, CuCl_2_ (0.1 mM) was added to 10^7^ cells for 48 h. Cell pellet was resuspended in HM buffer (0.25 M sucrose, 1 mM EDTA, 10 mM HEPES). Specimens were dried for 48 h at 65°C and dissolved in 300 µl of 65% nitric acid (Suprapure; Merck, Germany). Total amount of Cu was analyzed by AAS (Atomic absorption spectroscopy; Shimadzu AA6300, Japan).

### Statistical analysis

Statistical analysis was performed using SPSS 18.0 software. Data were analyzed by the Student's two tailed t test using Bonferroni correction for post-hoc pairwise comparisons or Kruskal-Wallis test. Data are given as mean ± SE.

## Results

### Generation of a human hepatoma ATP7B knockout cell line

**Figure 1:**
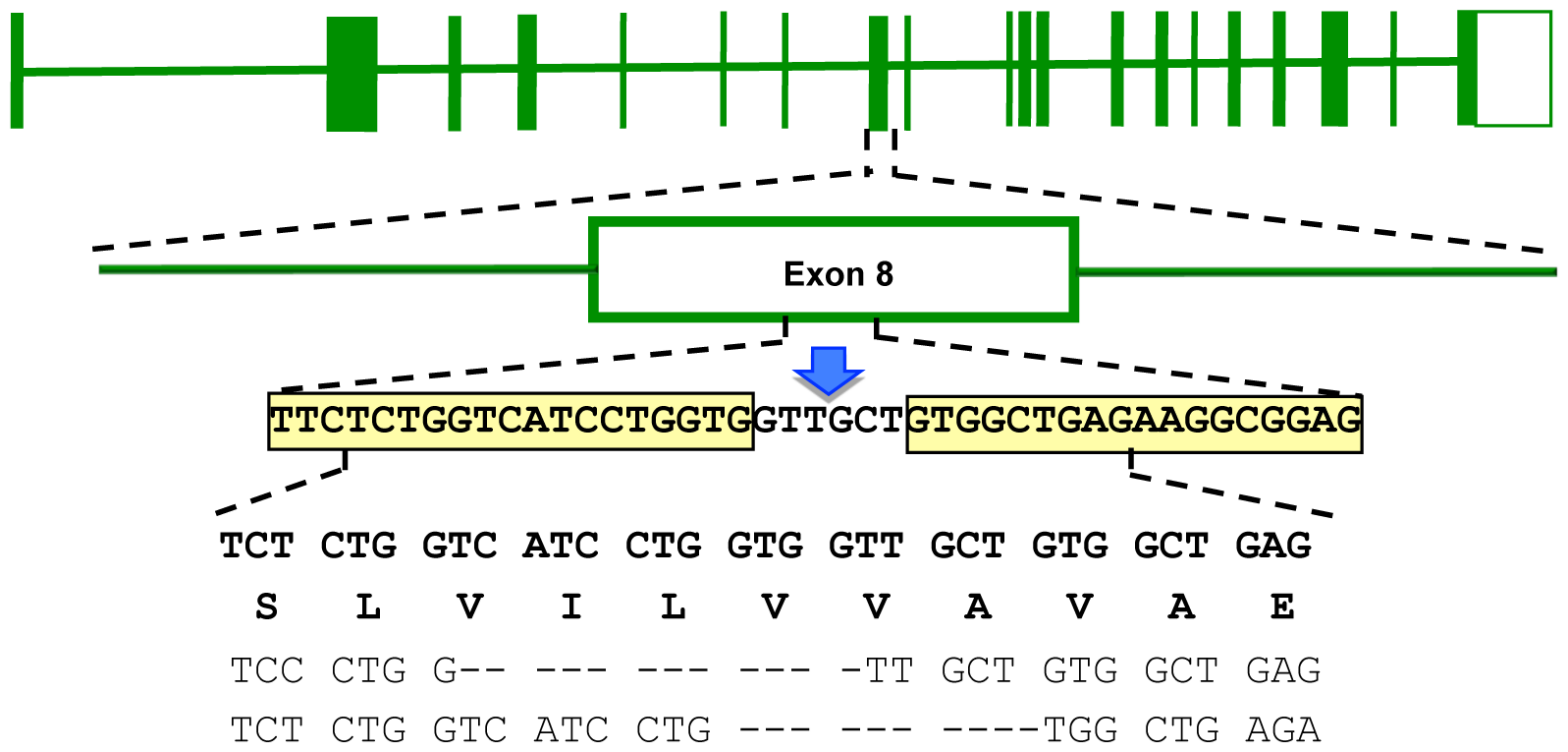
Genotype of the *ATP7B* KO cell line after ZFN directed mutagenesis. Top line shows the schematic structure of the entire WD gene (exons boxed). Middle lines represent a blow-up of exon 8 and specify the sequence of the proposed ZFN binding sites (boxed) and the *FOKI* restriction site (arrow). The four bottom lines specify the nucleotide sequences and codons of wild type (upper two lines) and the nucleotide sequence of both chromosomes observed in KO cell (last two lines). Dashes indicate the observed deletions.

We generated a stable human hepatoma ATP7B KO cell line to study Cu metabolism. HepG2 cells were transfected with plasmids encoding zinc-finger nuclease (ZFN) targeted to exon 8 of the ATP7B gene (Fig. 1). Sequence analysis of the genomic DNA revealed deletions around the FOKI cutting site and adjacent regions of the putative ZFN binding sites. Two deletions within exon 8 were found after cloning of individual sequences in E. coli. Sequence analysis of regions outside this area confirmed that other exons of ATP7B as well as exon/intron boundaries were identical to parental HepG2 cells.

**Figure 2:**
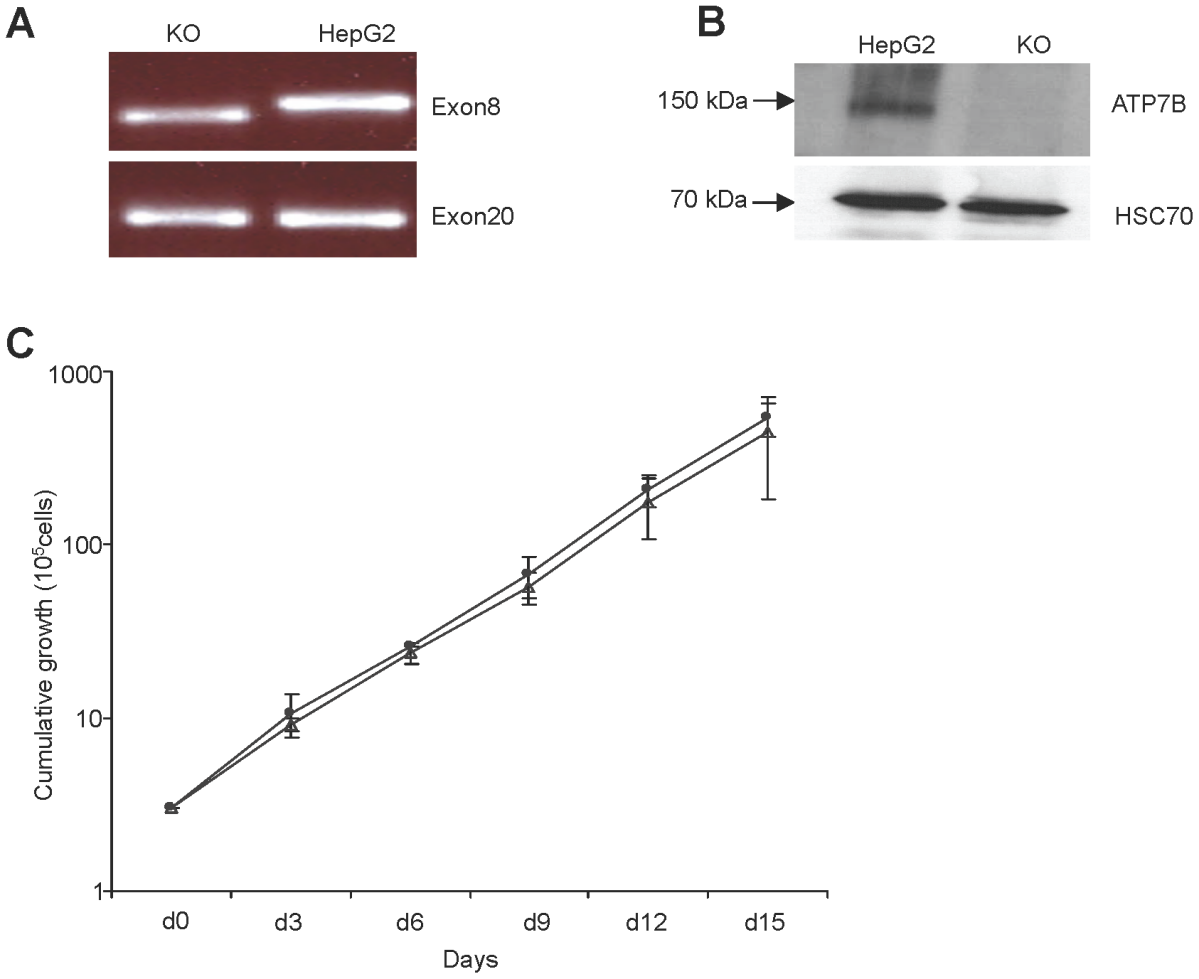
ATP7B cDNA, protein and growth characteristics of KO cell line. (A) PCR product of KO cell cDNA. Products cover the deletion in exon 8. Amplification of exon 20 served as control. (B) Western blot analysis of ATP7B protein and HSC70 loading control. (C) Growth curve of KO (closed circles) and HepG2 cells (open triangles). Cumulative growth is given. Asterisks indicate significance (p<0.05).

Analysis of the cDNA derived from KO cells by PCR analysis showed a deletion of exon 8 (Fig. 2A). Absence of ATP7B protein in KO cells as detected by Western blot suggests that the observed sequence aberrations are deleterious (Fig. 2B). Cell growth of the KO cells (Fig. 2C) revealed that the deletion within ATP7B does not impair the proliferation of the cells under standard cell culture conditions.

### Toxic impact of copper in human hepatoma ATP7B knockout cell line

**Figure 3:**
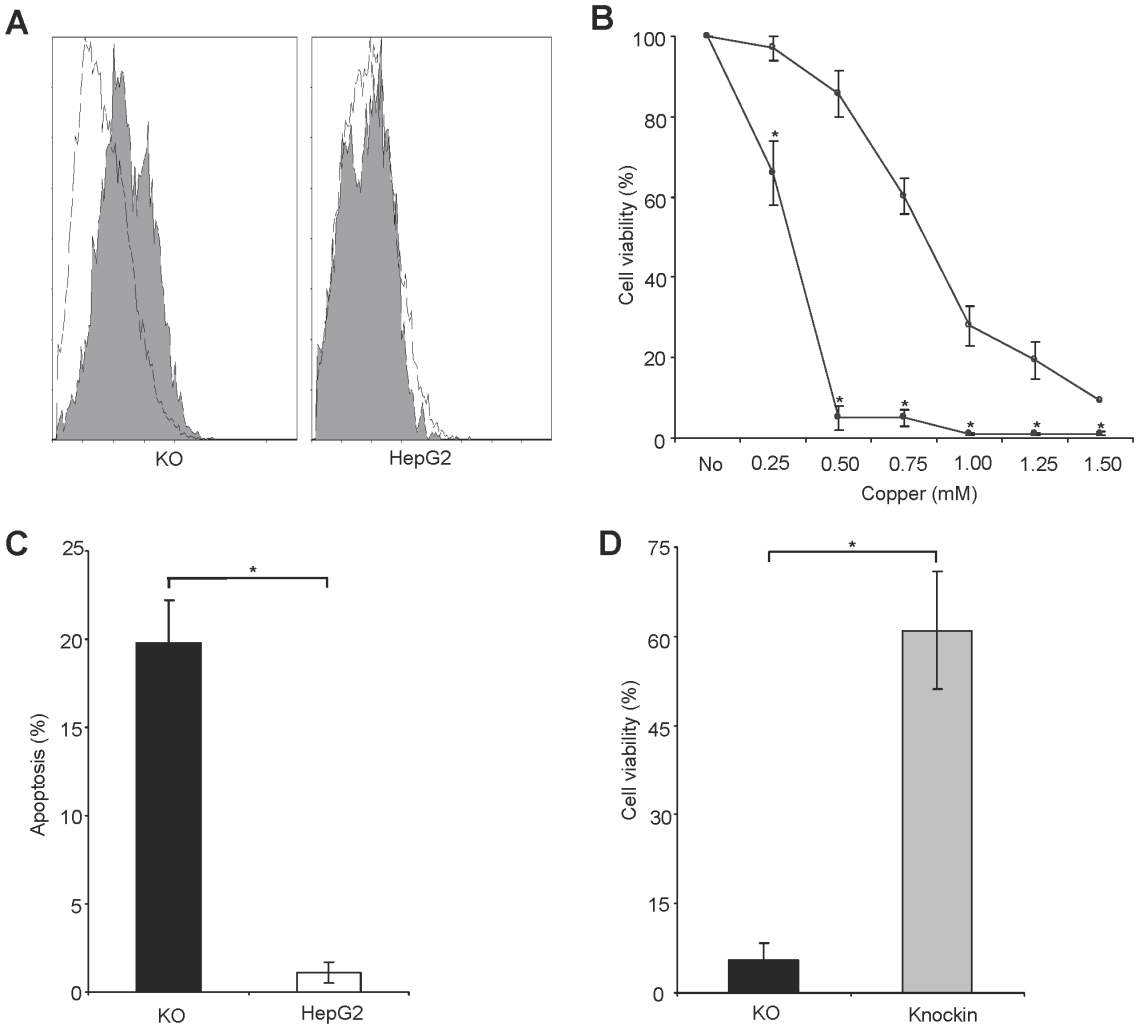
Oxidative stress and viability of KO cell line. (A) Oxidative stress following Cu exposure at 0.1 mM Cu for 1 h. The four histograms represent fluorescence of viable cells as obtained by flow cytometry after staining with H_2_DCFDA dye. A shift of the two histograms appears in KO cells after Cu exposure (shaded) relative to untreated control (open) indicating induction of OS. (B) Viability of KO (circles) and HepG2 cells (triangle) relative to untreated control was determined by MTT assay after 48 h of Cu exposure. (C) Induction of apoptosis was determined after 24 h of 0.1 mM Cu exposure using Annexin-V staining followed by flow cytometry analysis. (D) Cell viability of knockin versus KO cell is shown. Viability was determined at 0.5 mM Cu by MTT assay after 48 h. Data is represented as mean±SE of three independent experiments. Asterisks indicate significance (p<0.05).

The induction of oxidative stress (OS) was determined in KO cells using staining with H_2_DCFDA indicator dye followed by flow cytometry analysis. Addition of Cu to standard cell culture medium provoked a shift of histograms in KO cells but not in parental cells suggesting OS induction (Fig. 3A). Viability of KO cells was assessed in the presence of various Cu concentrations by MTT assay (Fig. 3B). KO cells rapidly (<24 h) underwent a significant change of cell morphology including cell rounding and shrinkage indicated by flow cytometry analysis (Figure S1). Significant apoptosis was induced in KO cells after Annexin-V staining (Fig. 3C). In order to confirm that the knockout is the cause of the observed Cu sensitivity, ATP7B was reintroduced in KO cells by lentiviral vector. The viability of KO cells in the presence of high Cu concentrations was restored to a similar range as observed in HepG2 cells (Fig. 3D). ATP7B knockin cells also restored resistance to Cu induced OS stress (data not shown).

### Copper loading of ATP7B knockout cells

**Figure 4:**
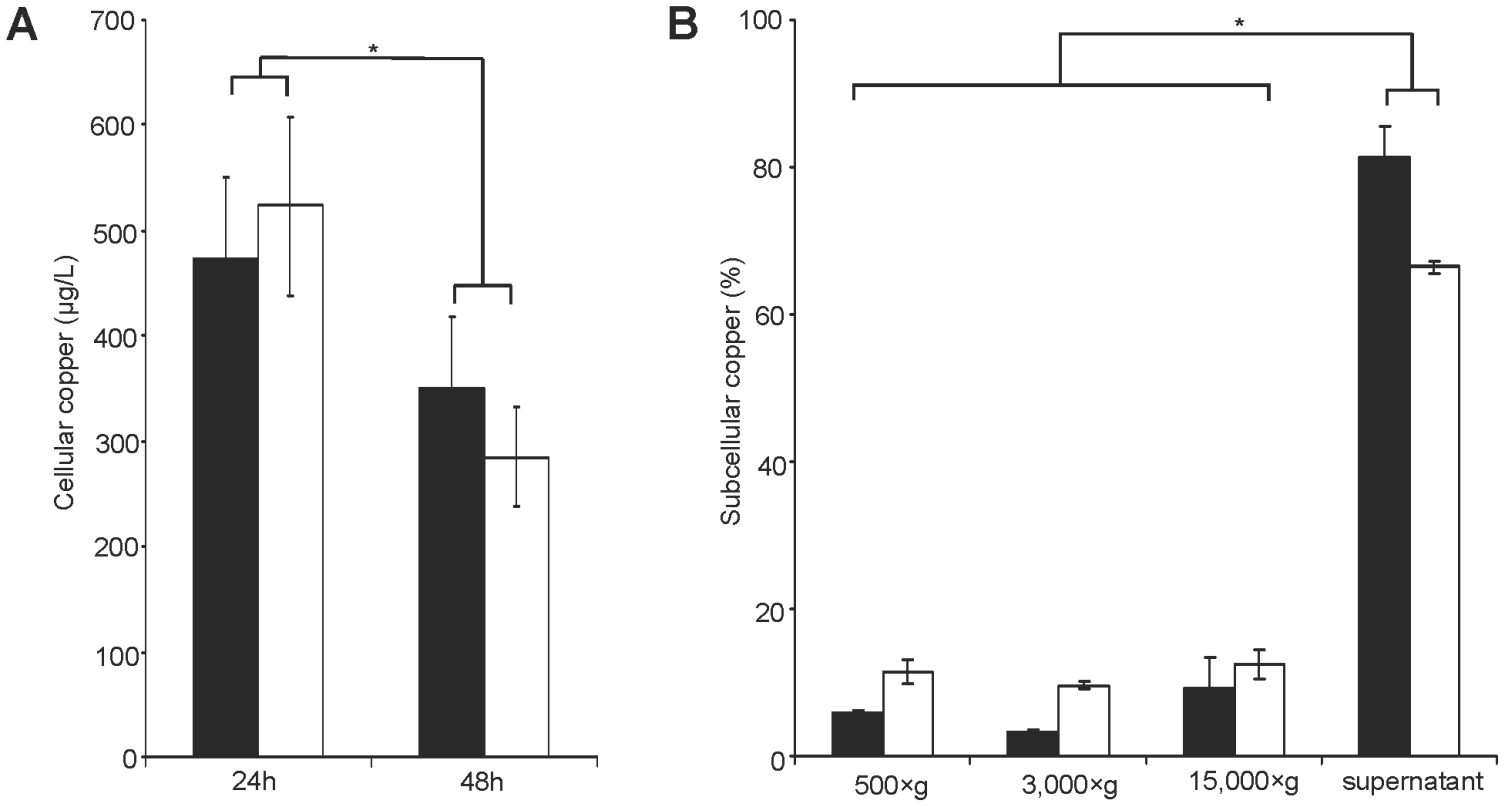
Cellular copper of KO cell line. (A) Total cellular Cu was determined after 24 h or after chase (48 h). Cells were subjected to AAS analysis. Data are recorded for viable 10^6^ cells. (B) Cu concentrations of cell lysates from KO (black) and HepG2 cells (open) are shown. Subcellular fractions were derived by differential centrifugation after 48 h Cu exposure. Data is represented as mean±SE of three independent experiments. Asterisks indicate significance (p<0.05).

The cellular Cu concentration was determined in KO and parental cells after Cu loading by AAS (Fig. 4A). Cellular Cu concentrations were almost identical in KO and HepG2 cells. The efflux of Cu from the cells was also assessed following a chase period consisting of several washings of the cells with standard cell culture media. Significant lower Cu concentrations were observed in both cell lines after the chase period. The subcellular Cu concentrations were analyzed after differential centrifugation. Most of the Cu was found in the supernatant of the 15,000xg fraction suggesting that Cu is mostly in the compartment enriched for cytosolic proteins (Fig. 4B). The observed differences between KO and HepG2 cells with regard to the subcellular Cu concentration did not reach significance.

### Increased copper tolerance of knockout cells following combined drug treatment

**Figure 5:**
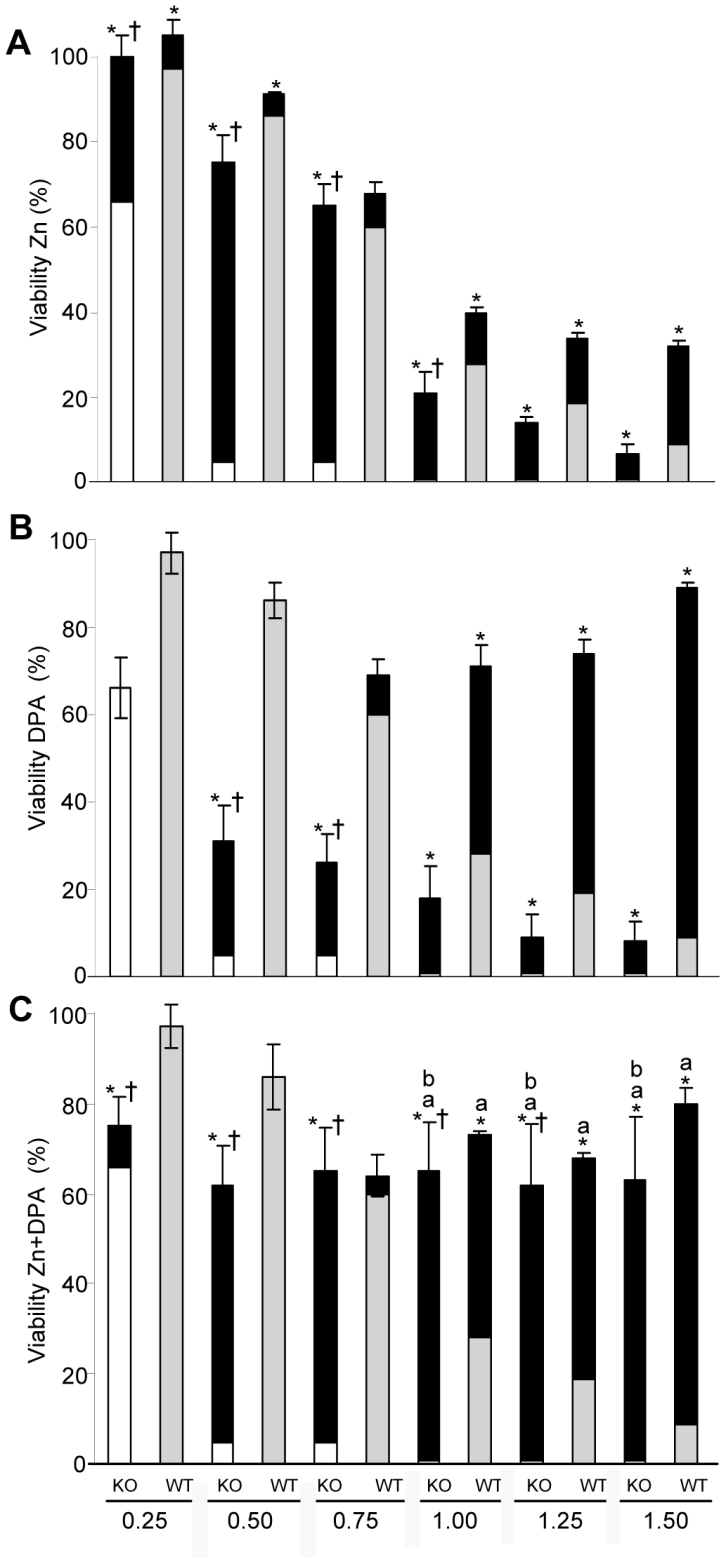
Drug induced tolerance of copper treated KO cells. Cells were treated with Zn, DPA and Zn+DPA and viability was determined by MTT assay. Zn pretreatment was for 2 h. DPA and Cu exposure was for 48 h. The gain of viability following treatment (black) is given relative to the viability of untreated KO (open) and HepG2 cells (shadow). Significance (p<0.05) is indicated: *treated vs untreated; ^†^gain by treatment in KO vs. gain in HepG2 cells; ^a^Zn+DPA treatment vs. Zn; ^b^Zn+DPA treatment vs. DPA.

The impact of zinc (Zn), D-penicillamine (DPA), and combined treatment (Zn+DPA) on the viability of the KO cells following Cu exposure was determined by MTT assay in the presence of different Cu concentrations (Fig. 5). Of note, Zn treatment (Fig. 5A) was reduced throughout the study to a 2 h pre-treatment period [12]. DPA was present for 48 h. After Zn treatment of KO cells, a high viability was gained (range 33.8% to 70.3%; mean 54.6±4%) up to a Cu concentration of 0.75 mM, whereas at higher Cu concentrations (≥ 1.0 mM) the improvement by Zn was lower (<20%) suggesting that a saturation of the effect is achieved. In HepG2 cells, the gain of viability by Zn treatment was generally lower (range 5.1% to 23.2%; mean 11.8±1%) suggesting that hepatocytes expressing ATP7B benefit less from zinc treatment. The effect of Zn treatment was not due to an overall higher toxicity of the KO cells, since HepG2 cells showed high rates of toxicity (>1.0 mM Cu) but relative low improvement. HepG2 cells had improved viability after DPA treatment (Fig. 5B) at Cu concentration ≥ 1.0 mM (range 43.3% to 80.1%; mean 59.3±3%). The effect of DPA on KO cells was significantly lower (range 0.2% to 25.9%; mean 13.2±6%) and further decreased (<10%) at higher Cu concentrations (>1.0 mM).Combined treatment (Zn+DPA) showed a highly synergistic effect in KO cells as compared to single drug treatment (Fig. 5C). Viability of KO cells was restored to high levels (mean 62.5±10%) at all Cu concentrations suggesting that combined Zn and DPA treatment has an advantage for the survival of human hepatocytes that lack ATP7B. HepG2 cells did not significantly improve viability by combined treatment as compared to single DPA treatment.

The effect of the drugs was also determined with respect to the induction of oxidative stress using H_2_DCFDA followed by flow cytometry analysis. While Zn and DPA treatment alone could not reduce values, the combined treatment (Zn+DPA) effectively reversed OS (Table S2).

### Cellular copper accumulation following drug treatment

**Figure 6:**
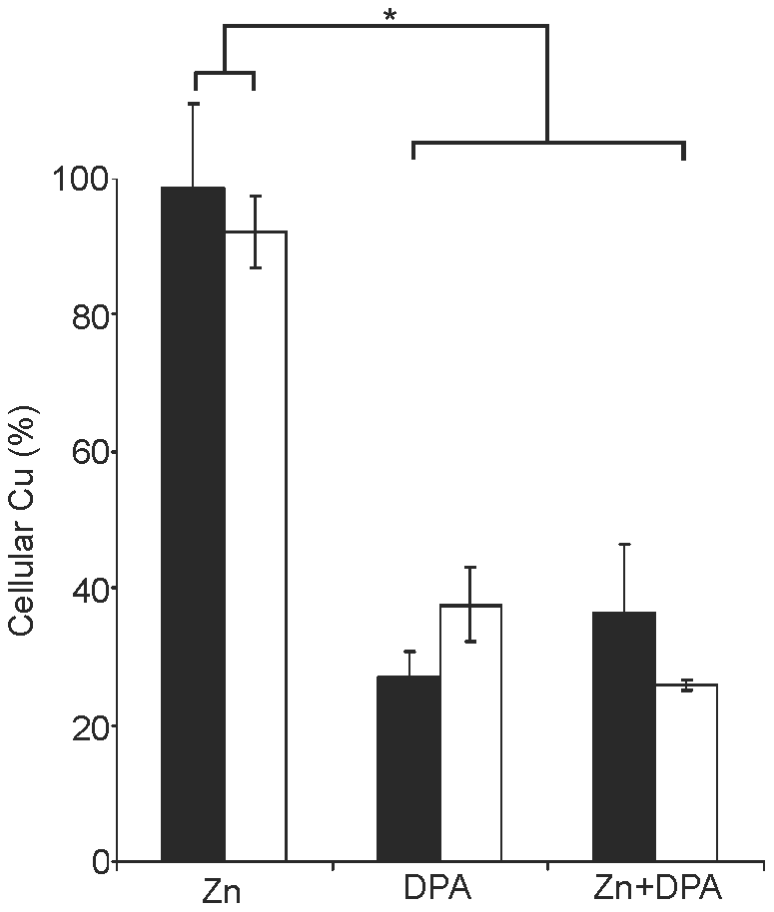
Cellular copper in KO cells after drug treatment. Cells were treated with drugs essentially as described for Figure 5. 0.1 mM Cu was used. Cells were subjected to Cu determination by AAS. Total cellular Cu of KO (black) and HepG2 cells (open) is given relative to the absolute number of the Cu concentration as shown in Figure 4A (100%). Data is represented as mean±SE of three independent experiments. Asterisks indicate significance (p<0.05).

The impact of Zn, DPA, and combined treatment (Zn+DPA) for cellular uptake of Cu was analysed by AAS. Essentially the same conditions as described for Fig. 5 were employed with the exception of a 0.1 mM Cu exposure, previously described to determine Cu concentrations in viable cells [11]. Higher Cu concentrations resulted in loss of viable cells. Zn treatment had no effect on the cellular Cu concentration in KO and HepG2 cells (Fig. 6). DPA and combined treatment (Zn+DPA) reduced cellular Cu concentrations to significant lower levels, confirming that the chelator is effective regardless of the presence or absence of ATP7B in human hepatocytes. Of note, the surplus effect of Zn observed in KO cells seems to be independent from the high cellular Cu concentrations observed in the cells.

### Induction of MT1X gene expression in knockout cells

Apart from intracellular Cu levels that were found to be similar in KO and parental cells (Fig. 6), a modulation of gene expression that regulate Cu uptake and/or oxidative stress may account for the observed protective effects of the drugs. Also, the increased viability of KO cells after Zn treatment was not revealed by a reduction of intracellular Cu concentrations (Fig 4A). Therefore, the mRNA expression of various marker genes was analyzed in presence/absence of the drugs. RNA was isolated after 12 h and subjected to real time PCR analysis. Under standard cell culture conditions, all genes assessed by real-time PCR analysis, including cytoprotective related HMOX1 gene and Cu binding *MT1X* gene [12], [23], showed similar levels of expression in both cells (Figure S2). Cells were incubated with Zn, DPA, and also with Cu which might modulate overall gene induction. A minor variance of gene expression (< fold factor 2) could be observed between the cells (Figure S3) except for *HMOX1* and *MT1X* expression.

**Figure 7:**
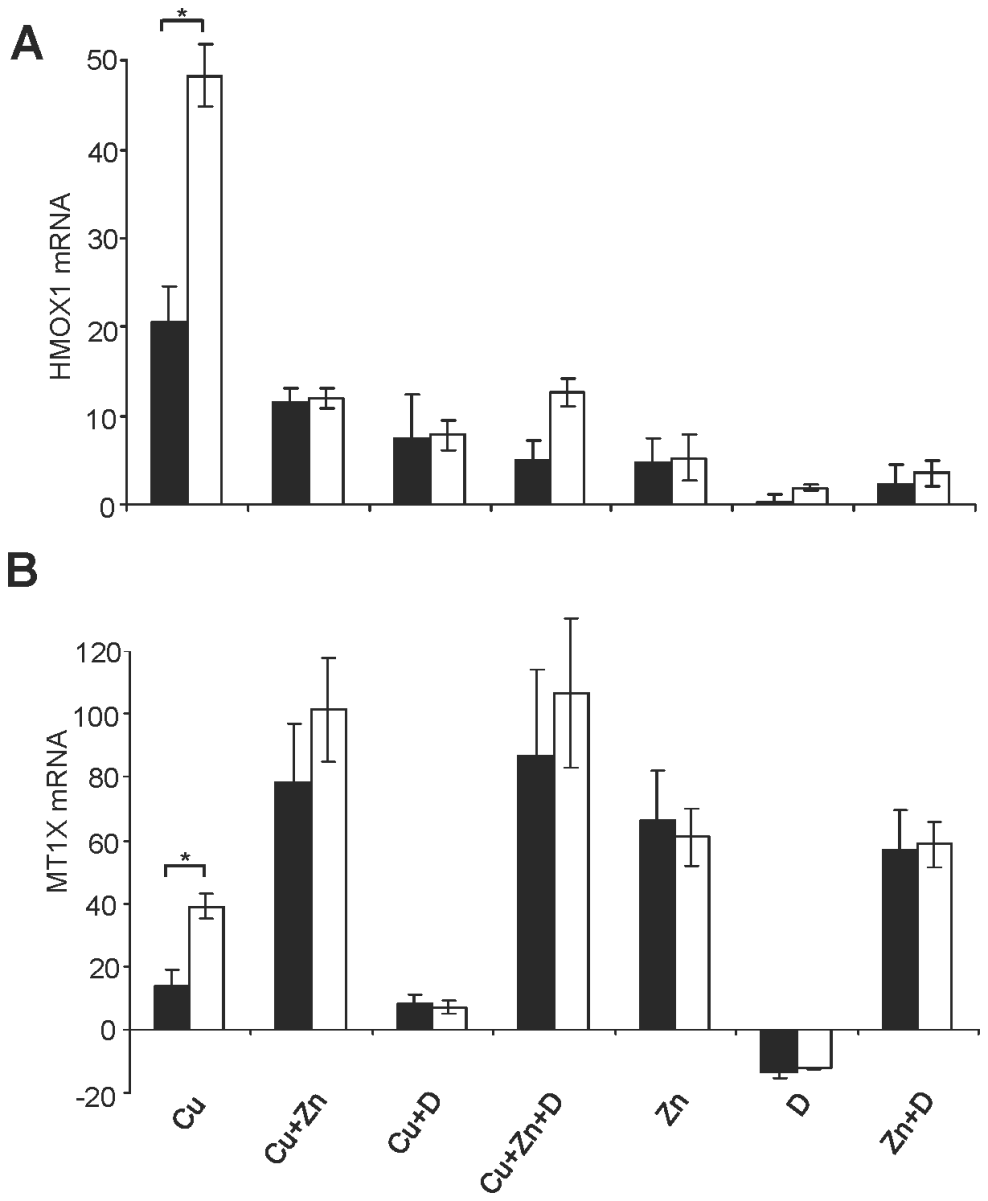
Gene expression of KO cells following drug treatment. Cells were treated with drugs for 2(Zn pretreatment) and 6 h (DPA). Total RNA was isolated after 6 h of treatment and subjected to reverse transcription followed by real-time PCR analysis. Gene expression of KO (black) and HepG2 cells (open) expressed as factor fold change relative to untreated is shown for *HMOX1* (A) and *MT1X* (B). Data is represented as mean±SE of three independent experiments. Asterisks indicate significance (p<0.05).

*HMOX1* gene expression (Fig. 7A) was highly induced after Cu exposure; however, KO cells showed a significant lower level as compared to HepG2 cells (fold factor 20.6±4 and 48.3±3, respectively). After drug treatment of the Cu exposed cells, *HMOX1* expression was significantly (p<0.05) reduced in both cells. Induction of *MT1X* gene expression following Cu exposure (Fig. 7B) was lower in KO cells as compared to HepG2 cells (fold factor 13.7±5 and 39.1±4, respectively). Of note, Zn treatment of KO cells highly upregulated MT1X expression to a similar level as observed in HepG2 cells (fold factor 78.3±18 and 101.1±16 respectively). *MT1X* gene expression was significantly (p<0.05) downregulated by DPA (fold factor of ≈13). Downregulation of *MT1X* was reversed by Cu exposure or Zn treatment.

## Discussion

To the best of our knowledge, we have generated for the first time a stable human KO hepatic cell line to explore the role of *ATP7B.* The KO cell line is suggested to represent a new cellular model of WD and can provide novel molecular insights into pathophysiology and treatment of the disease.

*ATP7B* knockout was achieved by ZFN directed mutagenesis that was reported to be highly specific [24]. Our data suggest that ZFN directed mutagenesis occurred in KO cells from at least two independent double strand breaks and non-homologous end-joining of the alleles confirming previous results from other cell lines. The targeted region in exon 8 of *ATP7B* encodes for the putative transmembrane domain TM3. Many missense mutations as well as deletions/insertions have been found in exon 8 of WD patients showing diverse phenotypes [25]. The mutations of KO cells resemble a compound heterozygous mutation (p.Val749fsX10 and p.V746_V750del) that is most likely associated with a severe phenotype as suggested by our in vitro assays. In contrast to hepatocytes, many non-hepatic cells, express the second common Cu transporter *ATP7A* [11], [26]. The KO cell line analyzed here provides insights into Cu homeostasis in the absence of the two main human transporters that excrete Cu.

KO cells could be highly passaged for more than a year without observing detrimental effects when standard cell culture conditions were used (data not shown). However, in the presence of low amounts of Cu, KO cells lost basic morphologic parameters within few hours. A high rate of oxidative stress and apoptosis were induced after Cu addition. Without additional Cu supplementation to the culture media, our real-time PCR analysis showed almost identical levels of gene expression in KO and parental cells. The *HMOX1* gene of KO cells was found with a lower expression level after Cu exposure. In contrast, high expression of *HMOX1* was observed in liver of LEC rats at the stage of hepatoma [23] suggesting that Cu exposure times and/or inflammation signals might play an additional role for *HMOX1* gene induction observed *in vivo.* Notably, ceruloplasmin synthesis, that is impaired after *ATP7B* malfunction [8], links Cu to iron homeostasis indicating that lower *HMOX1* expression could be related to altered heme, a potent inducer of *HMOX1.* However, understanding the molecular events between *ATP7B* impairment and *HMOX1* expression needs further work.

Although *ATP7B* was implicated to transport Cu out of the cells, the observed Cu efflux was almost as high as in the parental cell line suggesting that other Cu transporters or unspecific Cu loss may decrease cellular Cu. Our study on Cu efflux is, however, limited since HepG2 cells do not form complex liver architecture, although functions of polarized cells could be detected [27]. Low Cu concentrations were observed in subcellular fractions; however, differences with respect to the presence/absence of *ATP7B* were not noted [28]. *In vivo*, chronic Cu exposure of liver which might affect subcellular Cu storage as well as gene expression [28], [29] was not assessed in our short time analysis of tissue culture cells. The absolute concentrations found in KO cells resembled those observed in animal models of WD after a few months of Cu intake [28], [30]. Most subcellular Cu was found in the cytosolic compartment as observed previously [13], [28]. Importantly, after exposure of KO cells to Cu, a significantly lower *MT1X* expression was found suggesting that a portion of the Cu is either free or bound to other proteins. *MT1X* induction was suggested to represent a major natural defense mechanism to compensate for toxic metal concentrations [13], [17]. Our findings suggest that hepatic cells which lack *ATP7B* may have a lower capability to compensate for the toxic intracellular Cu.

Zinc has been used for treatment of WD during maintenance therapy after significant decoppering was achieved by chelators or in the presymptomatic phase of the disease [31]. The main mechanism underlying Zn therapy was proposed by the induction of *MT1X* expression in enterocytes, an effect cumulating in the excretion of the intestinal Cu by feces. Concomitantly after Zn treatment, low Cu was found in liver and other organs [32], [33]. Zinc was also shown to induce *MT1X* expression in hepatic cells [12], [32]. However, the molecular impact of Zn on human liver during treatment is poorly understood. Our data suggest that Zn treatment results in increased viability of hepatic cells that lack *ATP7B* expression (Fig. 5A). While basal expression of *MT1X* is similar in KO and parental cells (Fig. S2) and Zn does not reduce intracellular Cu load (Fig. 4A), induction of *MT1X* by Cu is impaired in KO cells (Fig. 7B) probably due to a role of *ATP7B* in processing Cu for activation of the *MT1X* promoter. As a result, hepatocytes that lack functional *ATP7B* may have a lower *MT1X*-mediated capability to evade toxic Cu unless Zn treatment is initiated. Of note, high intracellular Cu was found in KO cells after Zn treatment suggesting that while Zn does not block uptake of Cu, the intracellular Cu is compensated by *MT1X* induction. The effect of Zn on KO cells was found to be exhausted at higher Cu concentrations indicating that the therapeutic value of Zn treatment for increased survival of hepatocytes that lack *ATP7B* might be limited.

DPA is the most widely used drug for therapy of WD and long lasting clinical evidence suggests that DPA enhances Cu excretion via urine and results in the removal of Cu from tissues [31]. *In vitro* studies indicated that DPA is not accumulated within cells and may not reverse existing Cu levels [34], [35]. The latter finding is however challenged by others [36], [37]. As generation of hydrogen peroxide and other reactive oxygen species (ROS) by DPA have been shown and the implication of secondary mechanism other than chelating, e.g. by induction of metallothionein gene expression, have been observed [38], the exact mechanism of DPA for WD therapy remains uncertain. The results suggest that DPA alone downregulates *MT1X* expression, a finding that is controversial [39], [40]. Our data confirm previous data from rats suggesting that DPA is a repressor of *MT1X*. The effect of DPA on the molecular level of *MT1X* expression has, however, to be further investigated and could be related to decreased intracellular levels of Zn. We show that DPA is highly effective in blocking Cu accumulation in the absence of *ATP7B* underlining the importance of DPA for treatment of WD. However, in our study rescue from Cu induced toxicity was higher when *ATP7B* was expressed suggesting that a residual function of *ATP7B* that was observed for various mutations [41] might improve overall efficacy of DPA treatment.

Within the limitations of our *in vitro* study that does not comprise the constraints of the *in vivo* situation, e.g. drug availability in liver, the data suggest that a combined treatment by Zn and DPA has a synergistic effect on the viability of hepatocytes that lack *ATP7B*. Hepatic cells are shown to have a higher protection against oxidative stress and cell death when reduction of extracellular Cu via DPA is combined with the Zn-mediated induction of the *MT1X* gene. In the clinic, treatment by Zn and DPA is segregated in a timely fashion since DPA is known to chelate Zn thereby reducing the efficacy of both drugs [42]. Current developments in (i) the synthesis of new chelator molecules that have a higher specificity for Cu as opposed to Zn and (ii) in the induction of *MT1X* by alternate drugs that are not chelated by DPA [35], [43] may have a great potential to ameliorate the survival of hepatocytes lacking *ATP7B* and improve overall efficacy of the therapy in patients having WD.

## Supporting Information

Figure S1 **Cell size and granularity in KO cells following treatment with copper**. Cells were treated with 20 µM copper for 24 h. Cells were collected and analyzed by flow cytometry. Forward scatter (FSC) and side scatter (SSC) were determined. The percentage of cells in the ungated region is noted. Values are represented as mean±SE of three independent experiments.
(DOC)

Figure S2 **Gene expression of KO cells relative to HepG2**. Cells were cultivated using standard cell culture conditions. mRNA was isolated and subjected to real time PCR analysis using GAPDH gene for normalization. Fold change was calculated by ΔΔct method relative to HepG2 cells. Data is represented as mean±SE of three independent experiments. Note, that mean of fold change was below factor 3.
(DOC)

Figure S3 **Induction of gene expression in KO cells.** Cells were cultivated in medium containing copper, Zn and/or DPA for 6 h. mRNA was isolated and subjected to real time PCR analysis using the GAPDH gene for normalization. Fold change was calculated by ΔΔct method. Data is represented as mean±SE of three independent experiments. Note, that mean of fold change was below factor 2.
(DOC)

Table S1 **Primers used for qPCR**.
(DOC)

Table S2 **Effect of combined drug treatment for induction of oxidative stress.**
(DOC)
