# Supplementary figures and images for: The Effect of Zinc and D-Penicillamine in a Stable Human Hepatoma ATP7B Knockout Cell Line
Source: PLoS One. 2014 Jun 3;9(6):e98809. doi: 10.1371/journal.pone.0098809 (PMC4044041; doi:10.1371/journal.pone.0098809)

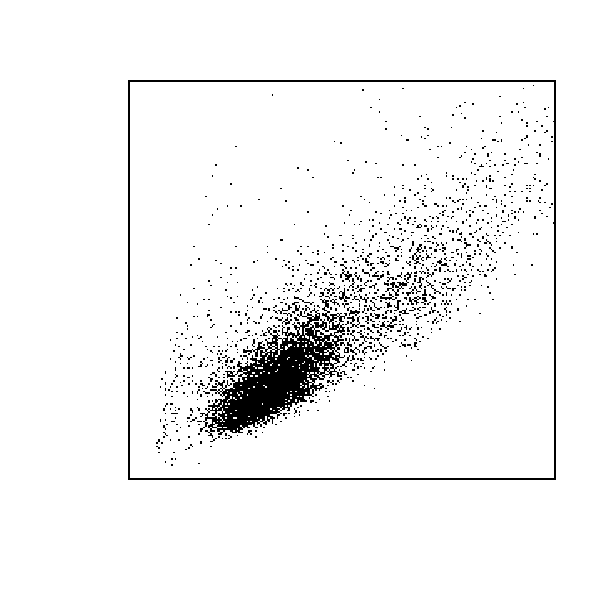

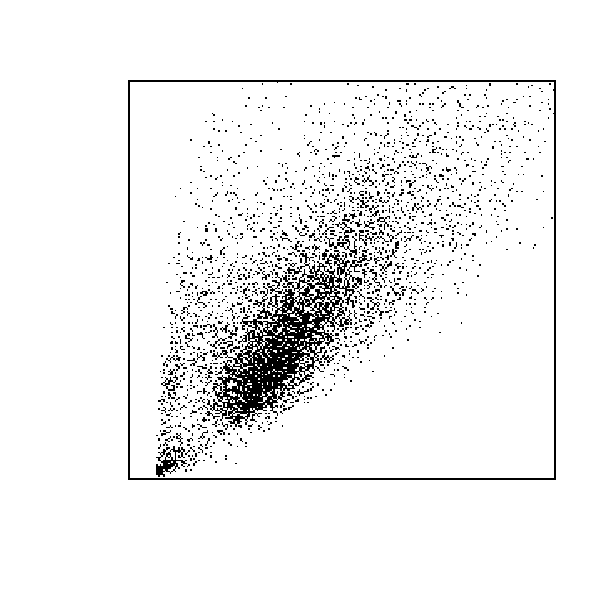


KO

HepG2

FSC

SSC

18.2±2.2

5.7±1.1

**Figure S1**

Supplement: Figure S1 — Cell size and granularity in KO cells following treatment with copper. Cells were treated with 20 µM copper for 24 h. Cells were collected and analyzed by flow cytometry. Forward scatter (FSC) and side scatter (SSC) were determined. The percentage of cells in the ungated region is noted. Values are represented as mean±SE of three independent experiments. (DOC) [file pone.0098809.s001.doc]
