# Supplementary material for: The Effect of Zinc and D-Penicillamine in a Stable Human Hepatoma ATP7B Knockout Cell Line
Source: PLoS One. 2014 Jun 3;9(6):e98809. doi: 10.1371/journal.pone.0098809 (PMC4044041; doi:10.1371/journal.pone.0098809)
Supplement: Figure S2 — Gene expression of KO cells relative to HepG2. Cells were cultivated using standard cell culture conditions. mRNA was isolated and subjected to real time PCR analysis using GAPDH gene for normalization. Fold change was calculated by ΔΔct method relative to HepG2 cells. Data is represented as mean±SE of three independent experiments. Note, that mean of fold change was below factor 3. (DOC) [file pone.0098809.s002.doc]

*SREBF1*

*SOD*

*p21*

*MT1X*

*ICAM-1*

*HNF1*

*HMOX1*

*GSTM1*

*GSS*

*GS*

*GRD1*

*G6PDH*

*GCKR*

*DMT1*

*CYP3A5*

*CX32*

*CTR2*

*CTR1*

*CP*

*COX17*

*COX4I1*

*COMMD1*

*MET*

*KRT19*

*KRT18*

*KRT8*

*KRT7*

*CCND3*

*CAT*

*ATOX1*

*AFP*

0

5

10

-5

-10

**Figure S2**
