# Supplementary material for: The Effect of Zinc and D-Penicillamine in a Stable Human Hepatoma ATP7B Knockout Cell Line
Source: PLoS One. 2014 Jun 3;9(6):e98809. doi: 10.1371/journal.pone.0098809 (PMC4044041; doi:10.1371/journal.pone.0098809)
Supplement: Figure S3 — Induction of gene expression in KO cells. Cells were cultivated in medium containing copper, Zn and/or DPA for 6 h. mRNA was isolated and subjected to real time PCR analysis using the GAPDH gene for normalization. Fold change was calculated by ΔΔct method. Data is represented as mean±SE of three independent experiments. Note, that mean of fold change was below factor 2. (DOC) [file pone.0098809.s003.doc]

KO

10

8

6

Fold change

4

2

0

-2

-4

*ATOX1 COMMD1 CTR1 CTR2 DMT1 SOD*

-6

-8

-10

HepG2

10

8

6

4

Fold change

2

0

-2

-4

*ATOX1 ATP7B COMMD1 CTR1 CTR2 DMT1 SOD*

-6

-8

-10

**Figure S3**
