# Supplementary material for: The Effect of Zinc and D-Penicillamine in a Stable Human Hepatoma ATP7B Knockout Cell Line
Source: PLoS One. 2014 Jun 3;9(6):e98809. doi: 10.1371/journal.pone.0098809 (PMC4044041; doi:10.1371/journal.pone.0098809)
Supplement: Table S1 — Primers used for qPCR. (DOC) [file pone.0098809.s004.doc]

**Table S1** **Primers used for qPCR**

| **Gene Symbol** | **Synonym** | **Accession Number** | **Primer (forward/reverse)** |
| --- | --- | --- | --- |
| *AFP* | Alpha-fetoprotein | BC027881 | CAGGAAGTCTGCTTTGCTGAAG/  TCACACCGAATGAAAGACTCG |
| *ATOX1* | ATX1 antioxidant protein 1 homolog | NM_004045 | CTGTGGAGGCTGTGCTGAAG/  TCTTGTTGGGCAGGTCAATG |
| *CAT* | Catalase | NM_001752 | AACGTCTGTGTGAGAACATTGC/  ATGTGGCTCCCGTAGTCAGG |
| *CCND3* | Cyclin D3 | NM_001136126 | AGGGATCACTGGCACTGAAG/  AGCTGGTCTGAGAGGCTTCC |
| *KRT7* | Keratin 7 | NM_005556 | TGGGAGCCGTGAATATCTCTG/  GCACTGCTGGAGAAGCTCAG |
| *KRT8* | Keratin 8 | NM_002273 | GGCTCCAGGCTGAGATTGAG/ GCATCCTTAATGGCCAGCTC |
| *KRT18* | Keratin 18 | X12881 | AGGTCAAGCTGGAGGCTGAG/  TTTGGATGGTTTGCATGGAG |
| *KRT19* | Keratin 19 | NM_002276 | AGGAGATTGCCACCTACCG/  AGAGGACCTTGGAGGCAGAC |
| *MET* | Met proto-oncogene | NM_000245 | AACCCGAATACTGCCCAGAC/ TATCCGGGACACCAGTTCAG |
| *COMMD1* | Copper metabolism domain containing 1 | NM_152516 | GGCAAATATGGACAGGAATCTG/  TGACAGCGTCTTCAGAATTTGG |
| *COX4I1* | Cytochrome c oxidase subunit IV isoform 1 | NM_001861 | TGGCAAGCGAGCAATTTCCAC/ GGTCACGCCGATCCATATAAGC |
| *COX17* | Cytochrome c oxidase assembly homolog 17 | NM_005694 | AGGAGAAGAAGCCGCTGAAG/  GGCCTCAATTAGATGTCCACAG |
| *CP* | Ceruloplasmin | NM_000096 | CACGGCCATAGCTTCCAATAC/  CCAAATTCCAGGTGTTCTTGG |
| *CTR1* | Copper transporter 1 | NM_001859 | GTCCCAGGACCAAATGGAAC/  ACCACCTGGATGATGTGCAG |
| *CTR2* | Copper transporter 2 | NM_001860 | TGCAGGCTCAGATTCATTCC/  TGACCACCTGGATGACATGG |
| *CX32* | Connexin 32 | NM_000166 | CCCTGCACAGACATGAGACC/  CAAACCTGTCCAGTTCATCCTG |
| *CYP3A5* | Cytochrome P450 | NM_000777 | AGTTCCGCCCTGAAAGGTTC/  TCTGGGTCCAGTTCCAAAGG |
| *DMT1* | Divalent metal transporter 1 | NM_001174127 | GGGTTGGCAATGTTTGATTG/  GCGTCCATGGTGTTCAGAAG |
| *GAPDH* | Glyceraldehyde-3-phosphate dehydrogenase | NM_002046 | CCCACTCCTCCACCTTTGAC/  CCACCACCCTGTTGCTGTAG |
| *GCKR* | Glucokinase regulator | NM_001486 | CAGATGATATTCGGGCTGCTC/  CACCGGAATAGGAGGCTCAG |
| *G6PDH* | Glucose 1-dehydogenase | NM_004285 | GAGAGCTGCAGAAGCCAGAC/  CAGCGAAGGTTGTCAATGTG |
| *GRD1* | Mitochondrial glutathione reductase | AF228704 | CCGATGTATCACGCAGTTACC/  CCCTGCATATGGATCCCAAC |
| *GS* | Glutamine synthetase | BC011852 | GCTGCCATACCAACTTCAGC/ CGCTTGCTTAGTTTCTCAATGG |
| *GSS* | Glutathione synthetase | NM_000178 | CCTAGCCGGTTTGTGCTAAAG/  TTTCAGGGCCTGTACCATTTC |
| *GSTM1* | Glutathione S-transferase mu 1 | NM_000561 | CCGTATATTTGAGCCCAAGTGC/  TCTTCTCCAAGCCCTCAAAGC |
| *HNF1* | Hepatic nuclear factor 1 | NM_000545 | TCCCTGGGTCCTACGTTCAC/  CTGCCCATGCTGTTGATGAC |
| *HMOX1* | Heme oxygenase 1 | NM_002133 | AAGAGGCCAAGACTGCGTTC/  TCTGGTCCTTGGTGTCATGG |
| *ICAM-1* | Intercellular adhesion molecule-1 | J03132 | TGACCGTGAATGTGCTCTCC/  TTCCGCTGGCGGTTATAGAG |
| *MT1X* | Metallothionein 1X | NM_005952 | CTCCTTGCCTCGAAATGGAC/  GCATTTGCACTCTTTGCATTTG |
| *p21* | Cyclin-dependent kinase inhibitor 1A | NM_001220778 | GCAGACCAGCATGACAGATTTC/  CTTCCTGTGGGCGGATTAGG |
| *SOD1* | Superoxide dismutase 1 | NM_000454 | ACTCTCAGGAGACCATTGCATC/  AAACGACTTCCAGCGTTTCC |
| *SREBF1* | Sterol regulatory element binding transcription factor | NM_001005291 | GGAGAGCCTGTACAGCTTGG/  GGCTGGGTCACACAGTTCAG |
