# Supplementary material for: The Effect of Zinc and D-Penicillamine in a Stable Human Hepatoma ATP7B Knockout Cell Line
Source: PLoS One. 2014 Jun 3;9(6):e98809. doi: 10.1371/journal.pone.0098809 (PMC4044041; doi:10.1371/journal.pone.0098809)
Supplement: Table S2 — Effect of combined drug treatment for induction of oxidative stress. (DOC) [file pone.0098809.s005.doc]

**Table S2** **Effect of combined drug treatment for induction of oxidative stress**

| **Group** | **Untreated** | **Cu** | **Cu+Zn** | **Cu+DPA** | **Cu+Zn+DPA** |
| --- | --- | --- | --- | --- | --- |
| **KO** | 1.3±0.2 | 2.7±0.2^1^ | 2.5±0.4^1^ | 2.6±0.9^1^ | 1.5±0.4^2^ |
| **HepG2** | 1.1±0.1 | 1.0±0.1 | 1.2±0.2 | 1.0±0.1 | 0.8±0.1 |

Fluorescence intensity (X-mean) of 3 independent experiments is shown as mean±SE.

Note, at Cu concentration of 0.1 mM used in the experiments, HepG2 cells do not show induction of oxidative stress.

^1^ versus untreated (p<0.05)

^2^ versus single treatment (p<0.05)
